# Quantitative Trait Loci for Morphological Traits and their Association with Functional Genes in *Raphanus sativus*

**DOI:** 10.3389/fpls.2016.00255

**Published:** 2016-03-04

**Authors:** Xiaona Yu, Su Ryun Choi, Vignesh Dhandapani, Jana Jeevan Rameneni, Xiaonan Li, Wenxing Pang, Ji-Young Lee, Yong Pyo Lim

**Affiliations:** ^1^Molecular Genetics and Genomics Laboratory, Department of Horticulture, Chungnam National UniversityDaejeon, South Korea; ^2^School of Biological Sciences, College of Natural Science, Seoul National UniversitySeoul, South Korea

**Keywords:** quantitative trait loci (QTLs), morphological trait, candidate gene, comparative mapping

## Abstract

Identification of quantitative trait loci (QTLs) governing morphologically important traits enables to comprehend their potential genetic mechanisms in the genetic breeding program. In this study, we used 210 F_2_ populations derived from a cross between two radish inbred lines (*Raphanus sativus*) “835” and “B2,” including 258 SSR markers were used to detect QTLs for 11 morphological traits that related to whole plant, leaf, and root yield in 3 years of replicated field test. Total 55 QTLs were detected which were distributed on each linkage group of the *Raphanus* genome. Individual QTLs accounted for 2.69–12.6 of the LOD value, and 0.82–16.25% of phenotypic variation. Several genomic regions have multiple traits that clustered together, suggested the existence of pleiotropy linkage. Synteny analysis of the QTL regions with *A. thaliana* genome selected orthologous genes in radish. InDels and SNPs in the parental lines were detected in those regions by Illumina genome sequence. Five identified candidate gene-based markers were validated by co-mapping with underlying QTLs affecting different traits. Semi-quantitative reverse transcriptase PCR analysis showed the different expression levels of these five genes in parental lines. In addition, comparative QTL analysis with *B. rapa* revealed six common QTL regions and four key major evolutionarily conserved crucifer blocks (J, U, R, and W) harboring QTL for morphological traits. The QTL positions identified in this study will provide a valuable resource for identifying more functional genes when whole radish genome sequence is released. Candidate genes identified in this study that co-localized in QTL regions are expected to facilitate in radish breeding programs.

## Introduction

Radish (*Raphanus sativus*; 2*n* = 18) is a diploid plant with estimated genome size of 530 Mb (Marie and Brown, 1993). It is an economically important crop grown for its edible root and leaves. The species belongs to Brassicaceae, a family that includes several other important crops, such as Chinese cabbage, cabbage, and oilseed. Among these crops of Brassicaceae, radish is perhaps the least characterized in terms of the genetics basic that govern morphological yield traits. Morphological traits of roots and leaves are important characteristics for radish. In particular, the initiation and growth of storage roots determine root yield and quality (Reid and English, 2000). The development of the storage roots and leaves involves complex interactions among environmental, genetic, and physiological factors. The axis of radish roots that thicken and form succulent biomass is derived from two parts: the upper part originates from the hypocotyl, where the lower part is from the true root (Tsuro et al., 2008). Root shape and size change in the course of vegetative growth, and these aspects have been selected by breeders according to the demands of markets. Thus, morphological traits are key contributors to the storage root yield and quality.

In recent decades, use of diverse germplasms has enabled development of several molecular markers and genetic linkage maps for radish (Bett and Lydiate, 2003; Tsuro et al., 2008; Budahn et al., 2009; Li et al., 2011; Shirasawa et al., 2011). Quantitative trait loci (QTLs) for cadmium accumulation (Xu et al., 2012), disease resistance (Kamei et al., 2010; Yu et al., 2013), glucosinolates (Zou et al., 2013), and root morphology (Tsuro et al., 2008; Hashida et al., 2013) have been identified. In Brassicaceae, the complete genome sequences of *Arabidopsis thaliana* and *Brassica rapa* have been determined, and the function of many genes have been characterized. A wide range of root and leaf morphological variations are reported in *B. rapa* cultivars (Lou et al., 2007; Li et al., 2009, 2013) A synteny map between these species should offer a powerful tool for the identification of candidate genes following QTL analysis.

With the rapid development of next generation sequencing (NGS) technologies, sequence analyses of whole genomes for a large number of crop plants can be accomplished in a short time (Metzker, 2010). The draft sequences of the Japanese cultivar radish genome have been published (Kitashiba et al., 2014). They comprise a total of 402 Mb scaffold sequences, with 116 Mb of these assigned to chromosomes by incomplete genome assembly. The combined use of QTL mapping, which detects functional loci for traits of interest, and whole-genome sequence information will advantage for breeding high-quality crops and vegetables. Although several QTLs have been mapped for root morphology (Tsuro et al., 2008; Hashida et al., 2013), most genes underlying trait variation have not been identified. The genome-wide scanning candidate gene approach usually locates chromosomal regions of QTLs with the DNA markers at the genetic distance level, which comprises the mass of candidate genes.

In this study, mapping populations previously used to examine the genetic control of fusarium wilt traits (Yu et al., 2013) were used to identify QTLs governing leaf, root, and whole plant characteristics in radish. QTLs were analyzed for those traits and candidate genes inferred from corresponding syntenic regions of *A. thaliana*. Using whole-genome NGS data of the parental lines in combination with comparative alignment with the *A. thaliana* genome allowed potential candidate genes with In/Del or SNP variations to be identified. Additionally, four conserved chromosomal blocks are identified by comparative analysis of *B. rapa* QTL regions, and structural and functional conservation between the radish genome and *Brassica* species are confirmed.

## Materials and methods

### Plant materials and growing conditions

A 210 F_2_ population derived from crossing inbred radish lines “835” and “B2,” which was previously used for developing the linkage map and QTL evaluation for the fusarium wilt disease resistant trait was used in this study (Yu et al., 2013). In total, 258 markers were used in this research, including newly developed EST-SSR reference markers (Shirasawa et al., 2011), and auxin, cytokinin and growth factor morphological-related functional markers. The two inbred lines showed distinct morphological characteristics: “835” showed elliptic root, long leaf, and an umbrella shape of above-ground plant parts, while “B2” showed slender root, short leaf, and a funnel shape of above-ground plant parts. For phenotypic investigation, all F_3_ progeny derived from the same F_2_ plant belong to the same F_2:3_ family, the mean of F_3_ phenotypic value replacing an F_2_ plant, called the F_2:3_ design (Zhang and Xu, 2004), eight F_2:3_ plants were planted per year of replications were used for phenotypic data measurement and average value of each line was used for trait statistics and QTL analyses. Seeds were sown in the field of Chungnam National University, Daejeon, Korea in September to November in 2012, 2013, and 2014. Eleven morphological and yield component traits (Table 1) were recorded from the F_2:3_ mapping population and the parental lines.

**Table 1:** **Means, ranges, and broad heritabilities (H^2^) estimated for 11 traits in parental lines and F_2:3_ generation**.

**Trait**	**ABS**	**Mean of parental line**			**F**_**2:3**_ **population**		
		**Year**	**B2**	**835**	**Mean**	**Range**	**H**^**2**^
Plant weight (g)	PW	2012	650.00 ± 111.80^a^	1725.00 ± 781.00	1421.56 ± 493.89	460.00–3800.00	0.48 (0.28-0.53)^b^
		2013	552.10 ± 174.59	1483.33 ± 301.39	1372.96 ± 558.11	190.00–3120.00	
		2014	610.84 ± 98.10	2042.40 ± 419.93	1774.99 ± 675.64	520.00–3710.00	
Plant height (cm)	PL	2012	34.80 ± 2.95	49.50 ± 5.80	58.24 ± 9.79	34.00–81.25.00	0.35
		2013	40.50 ± 6.08	48.67 ± 1.53	51.37 ± 10.48	18.00–79.33	(0.32–0.75)
		2014	38.00 ± 3.98	54.20 ± 10.03	44.70 ± 6.89	30.00–86.00	
Angular divergence	AD	2012	47.40 ± 4.22	75.00 ± 3.56	74.87 ± 10.68	51.25–100.75	0.64
		2013	53.00 ± 7.02	76.33 ± 6.35	68.97 ± 11.31	46.33–99.60	(0.37–0.68)
		2014	50.80 ± 2.81	94.80 ± 13.14	73.01 ± 12.30	42.667–120.00	
Exsertion length (cm)	EL	2012	8.70 ± 0.67	10.50 ± 4.20	10.85 ± 3.58	3.10–20.63	0.23
		2013	12.50 ± 2.08	10.33 ± 0.58	11.08 ± 3.97	2.00–27.50	(0.01–0.45)
		2014	10.30 ± 1.96	11.20 ± 3.03	12.26 ± 4.19	5.00–23.00	
Leaf number	LN	2012	15.00 ± 1.87	18.25 ± 4.11	18.41 ± 4.88	8.75–44.67	0.33
		2013	19.50 ± 3.21	13.67 ± 1.53	23.54 ± 6.22	9.00–44.75	(0.13–0.35)
		2014	17.40 ± 2.10	17.80 ± 1.48	22.82 ± 5.92	14.00–59.00	
Leaf length (cm)	LL	2012	28.00 ± 1.22	42.75 ± 3.40	44.44 ± 6.32	21.50–70.25	0.56
		2013	25.75 ± 8.25	48.00 ± 1.73	42.16 ± 6.14	27.75–58.40	(0.26–0.58)
		2014	27.30 ± 3.14	52.00 ± 6.745	44.70 ± 6.90	30.00–86.00	
Leaf width (cm)	LW	2012	13.80 ± 0.84	20.00 ± 3.37	17.97 ± 2.45	9.00–25.00	0.42
		2013	13.80 ± 3.01	22.00 ± 3.46	17.84 ± 2.93	9.50–24.60	(0.23–0.45)
		2014	13.72 ± 0.654	22.50 ± 1.66	19.12 ± 2.93	12.00–30.00	
Root weight (g)	RW	2012	374.00 ± 62.29	1115.00 ± 670.00	988.58 ± 395.86	240.00–3000.00	0.37
		2013	345.35 ± 118.29	1066.67 ± 230.94	931.18 ± 440.08	60.00–2260.00	(0.14–0.39)
		2014	372.14 ± 48.38	1554.00 ± 438.78	1198.46 ± 561.98	110.00–2680.00	
Root length (cm)	RL	2012	19.60 ± 1.52	22.75 ± 4.03	25.15 ± 5.43	13.67–44.17	0.43
		2013	23.00 ± 3.06	21.33 ± 2.31	20.18 ± 5.59	7.50–36.15	(0.21–0.49)
		2014	21.20 ± 2.34	28.40 ± 6.07	24.01 ± 5.58	10.00–41.00	
Root diameter (cm)	RD	2012	7.00 ± 0.50	9.75 ± 2.78	9.32 ± 2.22	5.00–31.50	0.34
		2013	7.10 ± 0.81	10.17 ± 0.76	9.41 ± 2.15	4.00–22.00	(0.19–0.37)
		2014	7.14 ± 0.38	11.04 ± 1.08	9.68 ± 1.91	5.00–15.00	
Root shap	RS	2012	5.00 ± 0	7.00 ± 2.16	5.01 ± 1.83	1.75–10.00	0.36
		2013	5.00 ± 1.15	7.00 ± 0	5.87 ± 1.59	1.00–9.00	(0.18–0.46)
		2014	4.60 ± 0.52	6.40 ± 2.51	6.64 ± 1.28	3.00–10.00	

a Mean ± stander error.

b 95% confidence interval.

### Statistical analysis and QTL mapping

SPSS statistics software (SPSS, Inc., Chicago, IL, USA) was used for correlation coefficient analysis. Heritability was calculated according to the formula H^2^ = $\sigma_{\text{g}}^{2}$ /($\sigma_{\text{g}}^{2}$ + $\sigma_{\text{g}1}^{2}$ /n + $\sigma_{\text{e}}^{2}$ /nr), where $\sigma_{\text{g}}^{2}$ and $\sigma_{\text{g}1}^{2}$ are the variance of the genotype and the variance of the interactions between environment, $\sigma_{\text{e}}^{2}$ is the variance of the error term, r is the number of replicates of each environment, and n is the number of environments (Knapp et al., 1985; Li et al., 2007; Ding et al., 2012). The previously described genetic map was used for QTL mapping (Yu et al., 2013). WinQTLCart 2.5 software (http://statgen.ncsu.edu/qtlcart/WQTLCart.htm; Wang et al., 2006) was used to perform QTL analysis. The composite interval mapping function was run using Model 6, with four parameters for forward and backward stepwise regression, a 10 cM window size, five control markers, and a 1 cM step size.

### Comparative QTL map alignment between the radish and *A. thaliana* and *B. rapa*

To identify loci functionally conserved across *R. sativus, A. thaliana*, and *B. rapa* for morphological traits, comparative alignment of QTL maps was performed. The *B. rapa* QTL map described by Li et al. (2013) was used for the comparison. Synteny analysis and identification of homologous chromosomal segments and crucifer building blocks (as proposed by Schranz et al., 2006), were conducted following the methods described by Ramchiary et al. (2011). For *A. thaliana* syntenic regions corresponding to QTLs, loci defined in the *A. thaliana* genome were searched in the TAIR database (http://www.arabidopsis.org/) to identify related candidate genes.

### High-throughput sequence and identification of SNPs and indels in candidate gene

High-throughput paired-end whole-genome sequencing of two parents (“835” and “B2”) were performed using an Illumina Hiseq2000 sequencer with 500 bp insertions between reads. Retrieved raw reads were quality checked, trimmed, and polished using “fastqc” (http://www.bioinformatics.babraham.ac.uk/projects/fastqc/), “jellyfish” (Marcais and Kingsford, 2011), and “quake” (Kelley et al., 2010) software. Whole-genome contigs and scaffolds were assembled by SOAPdenovo (Luo et al., 2012). Further, scaffold sequences in the radish genome were inferred by BLASTN and BLAT using EST sequences from *R. sativus* (http://www.ncbi.nlm.nih.gov) and *A. thaliana* genome (TAIR, http://www.arabidopsis.org). Twenty-four crucifer building blocks (Schranz et al., 2006) were identified in the radish genome by comparison with *A. thaliana*; previously functionally characterized candidate genes (such as Auxin, Cytokinin, Gibberellin, Growth regulating factor) were selected from *A. thaliana*, and searched for orthologous genes in the radish genome in those blocks harboring important QTLs using a homology and synteny analysis (Supplementary Table 3). We identified those candidate genes harboring mutated sequences including both SNPs and InDels by megaBLAST (Camacho et al., 2009) analysis. Further, open reading frame (ORF) prediction was executed based on megaBLAST results and manual inspection was carried out on homologs in the QTL regions of two parents “835” and “B2,” and identified non-synonymous amino acids caused by SNPs and InDels. Gene-based primers were designed for SNPs and InDels validation (Supplementary Table 3). Amplified PCR products were cloned into pGEM-T Easy Vector (Promega, USA) according to the manufacturer's instructions, and at least one clone was sequenced two times (Macrogen, Seoul, Korea). Nucleotide sequences between the parental lines were then compared to confirm the sequence variations.

### Expression analysis of candidate genes

Semi-quantitative RT-PCR was used to valuate expression differences of five co-mapped candidate genes between the parental line “835” and “B2.” Primers were used as given in Supplementary Table 3. Total RNA was extracted from 20-day-old leaf samples, using the method described by Li et al. (2013). Two micrograms of total RNA from each sample was reverse-transcribed in a 20 ml reaction mixture using a reverse transcriptase kit (Promega, USA). The radish actin gene was used as a control and the primer sequences (Yang et al., 2008): forward 5′-ATCTTCATGCTGCTTGGTGC, reverse 5′-GCTGATGATATTCAACC. The PCR program was as follows: a denaturing step of 95°C for 5 min; 35 cycles of 94°C for 40 s, 55°C for 45 s, and 72°C for 45 s; and a final extension of 7 min at 72°C.

## Results

### The revised map

**Figure 1:**
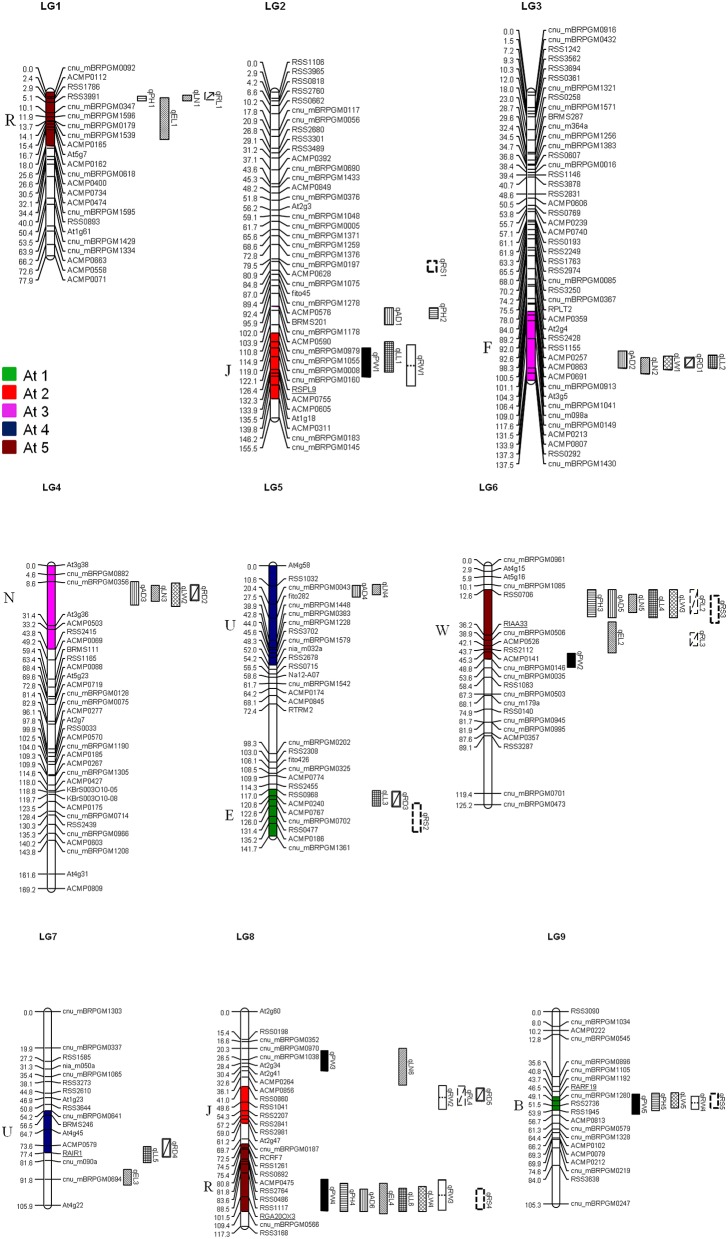
**Distribution of QTL for morphological and yield traits in the *R. sativus* genome**. QTL names are indicated by using the same abbreviations of trait names given in Table 1. Numbers in parenthesis indicate the year of QTL detection. The crucifer building blocks in each LG of *R. sativus* which are homologous to the five chromosomes of *A. thaliana* (At C1–At C5) are indicated by different colors.

We reviewed the quality of the raw genotype data from Yu et al. (2013) and identified 10 individuals and 13 markers which genotypes lose many data; these individuals and markers were deleted from our analysis. We constructed revised genetic maps for the radish genome base on adding 27 new genotype data from reference EST-SSR markers (Shirasawa et al., 2011), 16 SSR markers from *Brassica* species (Ramchiary et al., 2011; Li et al., 2013), and 8 candidate gene-based markers. (Supplementary Table 3) The nine linkage groups were named LG1–LG 9 following Shirasawa's map (2011) based on the common markers located on each chromosome. The revised maps incorporate total 258 markers and based on a total of 210 individuals. (Supplementary Table 1) Marker order changed in upper part of LG2 due to previously undetected errors in marker arrangement. In the updated map, LG5 and LG7 were joined named as LG5, in addition, LG8 was swapped as LG7 and more over we have got a new linkage group including 12 reference markers which was determined as LG8. (Figure 1, Supplementary Table 1).

### Phenotypic data analysis

The mean values of the two parental lines (“835” and “B2”), range and broad sense heritability (H^2^) values of F_2:3_ populations for 11 traits were summarized in Table 1. The parental lines were different for each characteristic, with the phenotypic values of “835” being higher than “B2” for all traits except exsertion length, leaf number and root length in 2013; heritability ranged from 0.23 for exsertion length to 0.64 for angular divergence. A wide range of variation was observed in the F_2:3_ population, with some traits showing significant transgressive segregation, e.g., plant height in 2012, leaf number in 2013 and 2014 (Table 1). The frequency distribution analysis for all traits in the mapping population showed continuous distribution in the 3-year replications, suggesting that multiple genes were involved for each trait (Supplementary Figure 1). Pearson's correlation coefficient analysis revealed moderate to strong positive correlations among leaf traits, root traits and whole plant traits. (Supplementary Table 2).

### QTL mapping and identification of crucifer building blocks

QTL mapping identified 55 loci related to morphology components distributed on each LG of the *Raphanus* genome. These QTLs accounted for 2.69–12.60 of the LOD value, 0.82–16.25% of phenotypic variation and the additive effect were range from −263.20 to 192.1. The putative QTLs for each trait identified in the F_2:3_ population are listed in Table 2, while the linkage map location of QTLs is depicted in Figure 1.

**Table 2:** **Position and effect of QTL for 11 morphological traits in *R. sativus***.

**Trait**	**QTL**	**Linkage group**	**Confidence interval (cM)**	**Marker interval**	**block region^a^**	**2012**			**2013**			**2014**		
						**LOD**	***R***^**2**^**(%)**	**Additive effects^b^**	**LOD**	***R***^**2**^**(%)**	**Additive effects**	**LOD**	***R***^**2**^**(%)**	**Additive effects**
Plant Weight	*q*PW1	LG2	122.2–136.0	cnu_mBRPGM0160-ACMP0311	J	3.10	3.68	192.10						
	*q*PW2	LG6	46.0–53.2	ACMP0141-cnu_mBRPGM0035	W				3.21	3.45	−216.20	3.40	3.85	−222.30
	*q*PW3	LG8	21.5–32.4	cnu_mBRPGM0970-ACMP0264	I				3.40	5.06	−170.30	3.23	4.28	−151.30
	*q*PW4	LG8	91.9–109.0	RSS1117-cnu_mBRPGM0566	R	8.20	8.25	−85.10	2.86	3.26	−37.80	6.10	4.85	−198.60
	*q*PW5	LG9	45.3–56.4	cnu_mBRPGM1192-ACMP0813	B	3.80	3.16	83.21						
Plant Height	*q*PH1	LG1	2.1–4.2	cnu_mBRPGM0092-RSS3991	R							3.10	3.25	−6.90
	*q*PH2	LG2	102.8–107.8	cnu_mBRPGM1178-cnu_mBRPGM0979		3.41	16.25	10.40	2.83	7.86	8.80			
	*q*PH3	LG6	12.7–26.7	RSS0706-cnu_mBRPGM0506	W	9.20	6.25	16.70	8.40	3.42	20.90	4.30	4.36	15.50
	*q*PH4	LG8	94.2–109.3	RSS1117-cnu_mBRPGM0566	R	6.60	7.26	−4.90	4.90	8.15	−9.10	3.90	6.22	−4.30
	*q*PH5	LG9	44.7–54.4	cnu_mBRPGM1192-ACMP0813	B	4.23	6.14	−1.10				3.90	4.86	−3.40
Angular Divergence	*q*AD1	LG2	103–110.9	ACMP0590-cnu_mBRPGM1055		3.00	5.27	9.50						
	*q*AD2	LG3	123.6–131.8	cnu_mBRPGM0149-ACMP0807	F	4.90	4.25	1.00	3.81	4.11	5.40			
	*q*AD3	LG4	8.5–20.7	cnu_mBRPGM0356-At3g36	N	5.70	3.15	2.60	3.50	4.45	3.60	3.90	6.25	4.20
	*q*AD4	LG5	10.4–16.6	RSS1032-cnu_mBRPGM0043	U	4.79	5.22	−3.90	3.80	6.13	−2.80			
	*q*AD5	LG6	12.8–27.3	RSS0706-cnu_mBRPGM0506	W				4.39	1.01	5.90	4.58	1.26	4.30
	*q*AD6	LG8	97.2–109.5	RSS1117-cnu_mBRPGM0566	R	7.30	5.89	−1.60	5.00	3.12	−2.20	3.90	2.11	−3.50
Exsertion Length	*q*EL1	LG1	2.8–22.4	ACMP0112-cnu_mBRPGM0618	R	4.10	13.15	2.70						
	*q*EL2	LG6	29.6–45.4	RSS0706-ACMP0141	W				6.20	6.12	2.30			
	*q*EL3	LG7	86.4–94.9	cnu_m090a-At4g22	U				6.10	3.11	1.90			
	*q*EL4	LG8	94.0–110.5	RSS1117-RSS3168	R	5.40	3.44	1.14	4.00	3.08	1.40	2.90	2.55	0.90
Leaf Number	*q*LN1	LG1	1.5–4.3	cnu_mBRPGM0092-RSS3991	R							3.20	8.12	−2.00
	*q*LN2	LG3	126.6–134.4	cnu_mBRPGM0149-RSS0292	F				6.60	2.15	−0.20	3.50	5.89	−0.40
	*q*LN3	LG4	10.3–18.9	cnu_mBRPGM0356-At3g36	N	6.10	4.86	−5.00	5.10	2.78	−0.80			
	*q*LN4	LG5	9.8–15.2	RSS1032-cnu_mBRPGM0043	U	4.80	6.14	−0.90						
	*q*LN5	LG6	14.8–24.5	RSS0706-cnu_mBRPGM0506	W				7.10	9.12	1.70	6.20	7.14	1.20
	*q*LN6	LG8	20.4–40.1	cnu_mBRPGM0970-RSS0860	I				4.70	6.12	−4.30	3.10	3.44	−1.90
Leaf Length	*q*LL1	LG2	119.0–133.6	cnu_mBRPGM0008-ACMP0605	J				3.10	4.80	5.00			
	*q*LL2	LG3	125.5–131.6	cnu_mBRPGM0149-ACMP0807	F	4.91	4.21	1.40	4.73	6.70	1.00	4.18	2.01	1.20
	*q*LL3	LG5	117.8–125.7	RSS0968-cnu_mBRPGM0702	E				3.90	9.22	2.80			
	*q*LL4	LG6	12.7–27.1	RSS0706-cnu_mBRPGM0506	W	5.71	2.04	1.80	5.08	4.30	2.90	5.14	3.77	2.10
	*q*LL5	LG7	73.8–82.8	ACMP0579-cnu_mBRPGM0694	U				3.94	5.89	1.50			
	*q*LL6	LG8	96.7–109.5	RSS1117-RSS3168	R	5.19	3.70	−1.20	3.87	3.45	−1.00	3.60	1.02	−3.00
Leaf Width	*q*LW1	LG3	125.9–132.3	cnu_mBRPGM0149-ACMP0807	F							4.45	2.15	0.70
	*q*LW2	LG4	9.0–21.3	cnu_mBRPGM0356-At3g36	N	6.50	5.21	2.60	3.97	6.12	1.60	4.60	3.46	1.30
	*q*LW3	LG6	12.7–27.3	RSS0706-cnu_mBRPGM0506	W	5.87	10.50	0.60	3.98	5.20	0.62			
	*q*LW4	LG8	95.5–109.6	RSS1117-RSS3168	R	5.93	4.21	−1.20	4.20	4.14	1.26	4.60	2.45	−1.70
	*q*LW5	LG9	44.2–52.7	cnu_mBRPGM1192-RSS1945	B	6.20	1.23	0.50				4.80	1.33	0.30
Root Weight	*q*RW1	LG2	120.5–140.1	cnu_mBRPGM0008-ACMP0311	J	4.80	14.22	178.10				3.10	4.26	114.70
	*q*RW2	LG8	40.5–53.7	ACMP0856-RSS2207	J							4.60	9.17	−263.20
	*q*RW3	LG8	92.3–108.5	RSS1117-cnu_mBRPGM0566	R	6.10	5.10	73.30	2.80	3.88	−31.59	3.10	2.14	−63.70
	*q*RW4	LG9	46.2–53.4	cnu_mBRPGM1192 -RSS1945	B	3.00	2.36	−49.60				2.80	3.70	−165.40
Root Length	*q*RL1	LG1	1.2–4.7	cnu_mBRPGM0092-RSS3991	R							2.69	8.57	4.10
	*q*RL2	LG6	12.8–25.7	RSS0706-RIAA33	W				3.20	4.15	1.10			
	*q*RL3	LG6	34.0–41.5	RSS0706-cnu_mBRPGM0506	W	7.20	4.25	0.40	7.00	5.22	0.90	6.20	4.82	3.00
	*q*RL4	LG8	40.6–51.7	ACMP0856-RSS2207	J				3.14	9.45	3.99			
Root Diameter	*q*RD1	LG3	126.2–131.0	cnu_mBRPGM0149-ACMP0213	F				5.15	3.40	−0.34	11.20	4.25	−3.00
	*q*RD2	LG4	10.8–18.6	cnu_mBRPGM0356-At3g36	N	12.60	9.50	−0.80	8.10	6.27	−0.38	6.10	4.72	−0.43
	*q*RD3	LG5	118.3–126.4	RSS0968-RSS0477	E							3.10	3.14	0.90
	*q*RD4	LG7	70.0–80.0	At4g45-cnu_m090a	U							3.00	2.89	0.26
	*q*RD5	LG8	42.6–49.2	RSS0860-RSS1041	J				3.00	4.21	2.86	3.11	0.82	3.77
Root Shape	*q*RS1	LG2	80.3–85.6	ACMP0628-fito45	A/M				3.90	9.22	0.37			
	*q*RS2	LG5	125.7–140.8	cnu_mBRPGM0702-cnu_mBRPGM1361	E	3.70	5.24	1.15						
	*q*RS3	LG6	15.3–29.3	RSS0706-cnu_mBRPGM0506	W				8.70	5.20	0.24	5.00	3.50	0.15
	*q*RS4	LG8	97.3–108.4	RSS1117-cnu_mBRPGM0566	R	4.35	3.77	−0.16	5.05	4.12	−0.32	3.49	3.10	−0.48
	*q*RS5	LG9	44.5–52.4	cnu_mBRPGM1192-RSS1945	B							3.02	1.25	−0.76

a Blocks expanded compared to the previous reports (Schranz et al., 2006).

b Positive value indicates that the allele from “B2” increases the value of the trait, and a negative value indicates that the “B2” allele reduces the trait value.

#### Whole plant traits

Five QTLs associated with plant weight (PW) were detected: two QTLs in LG8 and one QTL in each of LG 2, LG 6, and LG 9 (Table 2). *q*PW4 (R block) showed relatively higher LOD values and phenotypic variations was detected over the 3 years.

We identified five QTLs for plant height (PH) and distributed on LG1, LG2, LG6, LG8, and LG9. Two of these QTL (*q*PH2 and *q*PH5) were observed during 2 out of 3 years, while the *q*PH1 were detected in only 1 year.

Four QTLs were detected for exsertion length (EL), among which *q*EL1 in the LG1 and *q*EL2 in the LG6, *q*EL3 in the LG7, only *q*EL4 in the LG8 which was found consistently 3 years, in 2012, 2013, and 2014.

#### Leaf traits

Four leaf traits were used for QTL analysis: leaf length (LL), leaf width (LW), leaf number (LN), and angular divergence (AD) (Figure 1, Table 2).

Six QTLs were identified for LN. *q*LN1 in the LG1, *q*LN2 in the LG3, and *q*LN3 in the LG4, QTLs in LG5 (*q*LN4), LG7 (*q*LN5), and LG8 (*q*LN6) were detected in the U, W, and I blocks respectively. Six QTLs were identified to control LL. *q*LL2, *q*LL4, and *q*LL6 were found significant in all 3 years. However, *q*LL1, *q*LL3, and *q*LL5 were only detected in 2013.

For LW, five QTLs were detected, of which *q*LW1 in the F block of LG3, *q*LW2 in the N block of LG4, *q*LW3 in LG6, *q*LW4 in LG8, and *q*LW5 in LG9. Among them, only *q*LW2 and *q*LW4 were consistently found in 2012, 2013, and 2014.

Four QTLs located on LG1, 6, 7, and 8 were detected for AD, *q*AD6 in the R block of LG8 seemed to be the major QTL and was found in all 3 years.

#### Root traits

Four root traits were used for QTL analysis: root weight (RW), root length (RL), root diameter (RD), and root shape (RS; Figure 1, Table 2).

Four QTLs were detected for RW, these were distributed in four linkage groups: two on LG8 (*q*RW2 and *q*RW3), and one each on LG2 (*q*RW1), and LG9 (*q*RW4). Four and five QTLs were identified for RL and RD, respectively. Of these, *q*RL2 and *q*RL3 mapped to the W blocks of LG6 in different years, *q*RL1 mapped to the R block of LG1, *q*RL4 to the J block of LG8. For RD, *q*RD1 in the F block and qRD5 in the J block were detected in 2 of the 3 years, *q*RD2 was found in the N block and *q*RD3 and qRD4 detected in the E block of LG5 and U block of LG7.

Five QTLs associated with RS were detected in different linkage groups *q*RS1 (A/M block), *q*RS2 (E block), and *q*RS5 (B block) were only detected in a single year, *q*RS4 on LG8 (R block) were detected in 3 years.

### Sequence validation and expression analysis of functional candidate genes

Whole-genome sequencing of the “835” and “B2” parental line produced 3.3 (6.26X) and 3.5 (6.73X) giga base pairs (Gb) after trimming and polishing, respectively, and SOAPdenovo genome sequence assembly generated scaffolds of 264,564,155 and 276,810,577 bp. Then we designed a total of 50 gene-based markers from *A. thaliana* and aligned to the parental lines to validate the SNP and In/Dels experimentally (Supplementary Table 3). Five markers showed clear polymorphisms and co-localizations in map with QTL positions on their respective linkage groups (Figure 1). The marker associated squamosa promoter binding protein- like gene RSPL9 (AT2G42200.1) mapped to LG2, where QTLs include PW, LL, and RW. The auxin gene related markers RIAA33 (AT5G57420.1), RAIR1 (AT4G12550.1), and RARF19 (AT1G19220.1) mapped to LG6, LG7, and LG9, respectively. The gibberellin biosynthesis gene marker RGA20OX3 (AT5G07200.1) was located on LG8 associated with AD, LN, LL, LW and RD, RS.

**Figure 2:**
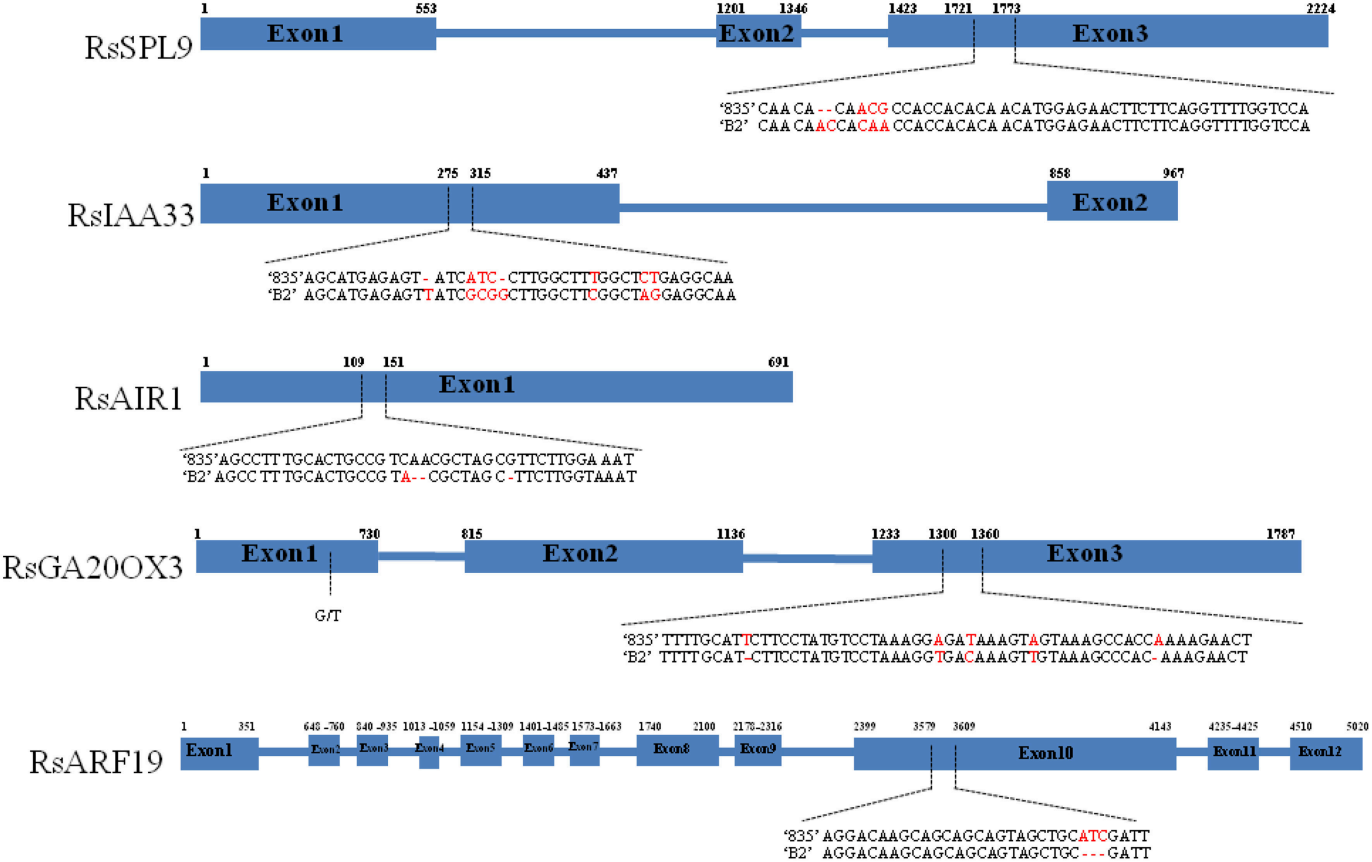
**Nucleotide polymorphisms of RsSPL9, RsIAA33, RsAIR1, RsGA20OX3, and RsARF19 between “835” and “B2.”** Blue boxes and blue lines indicate exons and introns, respectively. Dashed arrows indicate nucleotide variations. Numbers above the boxes indicate exon start and stop sites.

We resequenced the one of In/Dels regions to validate whether the aligned sequence data of the parental lines were correct. The sequences of RsSPL9, RsIAA33, RsAIR1, RsGA20OX3, and RsARF19 showed over 80% nucleotide identities with homologous sequences from *A. thaliana* (Figure 2). RsSPL9 contained a 2 bp insertion in “B2” and variations in the adjacent three nucleotides in exon 3. In RsIAA33, nucleotides changed in exon 1, and in RsAIR1 consisted of 2 bp deletions in “B2” in exon 1. RsGA20OX3 had five SNPs located closely together between “B2” and “835,” and RsARF19 had 3 bp insertions in “835.”

**Figure 3:**
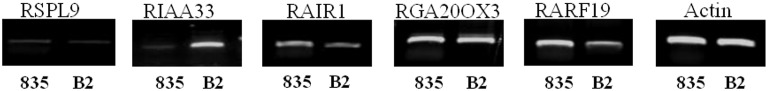
**Semi-quantitative RT-PCR analysis of candidate genes using RNA samples extracted from 20-day-old seedlings of “835” and “B2.”** Actin gene amplicons were used as control for RNA quantity.

Semi-quantitative RT-PCR analysis was used to evaluate expression differences of candidate genes between the parental lines. The RsIAA33, RsAIR1, and RsARF19 showed differential gene expression between “835” and “B2” lines (Figure 3). RsARF19 and RsAIR1 showed a little higher expression in “835,” noteworthy the RsIAA33, which showed strong expression in “B2” but little expression in “835.” RsGA20OX3 had strong expression, whereas, RsSPL9 was lowly expressed in both parents, respectively.

### Comparative QTL map alignment between *A. thaliana* and *B. rapa*

The comparative QTL map between *R. sativus* analyzed in this research with the *A. thaliana* genome and the *B. rapa* QTL map of Li et al. (2013) allowed us to identify the homological region of QTLs with different characteristics and the tight linkage of candidate genes conferring this traits. A total of six homologous groups were identified through the comparative alignment of the QTL maps between three species. Concomitant structural and functionally conserved loci were found in J, W, U, and R blocks (Figure 3). The first homologous group consisted of the R block of At5, LG1 of radish, and *B. rapa* A10; The second homologous group consisted of the J block of At2, LG2 of radish and *B. rapa* A5. The third homologous group consisted of the W block of At5, radish LG6, and *B. rapa* A10, and common QTL for leaf traits. The fourth homologous group consisted of radish LG7 and *B. rapa* A8, along with the U block in At4, these contained common traits of leaf length. The most important groups were J and R blocks located in LG8 which homology with *B. rapa* A3, and contained similar QTL traits for plant height in R block.

## Discussion

### Variation of phenotypic trait in parental lines and population

In the present study, we detected 55 QTLs for 11 agronomic traits using an F_2:3_ population from a cross of the inbred lines “835” and “B2” over 3 consecutive years. Because the F_2:3_ population consisted of genetic information from both heterozygous and homozygous individuals, it allowed the exploration of advantageous QTL/genes for radish breeding improvements. The map was mainly constructed based on SSR markers aligned with the high-density reference maps of Shirasawa et al. (2011) in radish, and Li et al. (2013) in *B. rapa*. It was easy to compare the positions of QTLs detected with those reported for corresponding morphological traits in other *Brassica* species (Li et al., 2013).

Transgressive segregation is defined as the individuals in segregating populations that fall beyond their parental phenotypes, and it was observed for EL in 2014 and LN in 2013 and 2014. The frequency distributions in F_2:3_ lines segregated continuously, our results were in agreement with the idea that morphologically traits were commonly controlled by multiple QTLs/genes that cause continuous phenotype variation in plants. Phenotypic correlations between whole plant, root and leaf traits showed significant positive influences in the 3 years, allowing co-mapping of different QTLs governing whole plant, leaf and root yield. Because we surveyed 3 years of phenotypes, and the genotypes were available, the broad-sense heritability (H^2^) was used measures the proportion of total phenotype that is affected by genetic and environmental variations. The traits AD and LL have relatively high broad sense heritability indicated the proportion of phenotypic variation which may be mainly due to genetic factors, moreover, the EL and RD traits are relatively low value that can be easily influenced by the environment (Table 1). On the other hand, narrow-sense heritability (h^2^) defined as the proportion of phenotypic variance that is due to additive genetic effects. The additive variance gives only limited information about the genetic model for QTLs infuencing the trait (Abney et al., 2001).

### QTL for morphological characters and plant growth

In this study, some traits, such as PH, PW, LL, LW RD, and RS were highly correlated (Supplementary Table 2), and were controlled by QTLs located on the same linkage groups: LG6 and LG8. Another same set of QTL map positions appeared to control LL and AD were located on LG2 and LG3. A similar phenomenon of a clustering of QTLs affecting different traits in the same genetic interval is observed in *B. oleracea* and *B. rapa* (Kennard et al., 1994; Song et al., 1995). The co-localization of QTL governing different traits in the same genetic interval is suggested to be due to either pleiotropy, with a single gene or a tight linkage of genetic loci that affects multiple characteristics, or that their inheritance is functionally linked by a common mechanistic basis (Xiao et al., 1996; Lou et al., 2007; Srinivas et al., 2009).

Some of the QTL traits showed low consistency over the 3 years. This is likely a result of environmental effects; it is expected that genotype-environment interactions generate inconsistency in plant development (Hashida et al., 2013). The values for the plant weight trait in 2013 were smaller than in the other 2 years (Table 1). The temperature was lower in October 2013 than in 2012 and 2014; differences in temperature during the primary growth of radish can lead to differences in nutrition accumulation required for growth (Radin, 1990; Tsuro et al., 2008). Plant shape (e.g., angular divergence) is an important trait for cultivation; it largely affects planting density and it effects a plant's reaction to light. This trait has been extensively studied in many crops (Pereira and Lee, 1995; Tian et al., 2011; Ding et al., 2012) but is less understood in radish. The *qAD*1, *qAD*2, *qAD*3, and *qAD*4 identified here contributed positively to the umbrella-shaped radish of the “835” allele, thus, increasing the size of the upper parts and the ability to absorb light. These QTLs could make a good genetic resource for the selection of high-quality yield in radish.

### Functional genes were detected in different QTL blocks of the radish genome

Through the whole-genome sequencing of the two parents, we directly analyzed the genomic regions of the QTLs that are homologous with *A. thaliana* and identified candidate genes are co-localized for each QTL of interest. These results indicate that next-generation sequencing technology is an efficient strategy for candidate gene identification and enhanced the efficiency of QTL mapping.

Previously, 24 defined crucifer building blocks in the *R. sativus* genome were compared with *A. thaliana* (Shirasawa et al., 2011), and characterized candidate genes were selected from *A. thaliana* and orthologous genes searched for in the *R. sativus* genome. The gene-based markers linked to stable QTLs could provide practical tools for selecting candidate genes in radish. Through the co-linearity of the genomic sequence and conservation of homolog gene, the gene-based markers were found either as QTLs or were closely linked with QTLs of different traits. There were three genes related to the auxin gene family; these regulated various aspects of plant growth and development. IAA33 mapped to LG6, and is involved in the auxin-activated signaling pathway and response to auxin, and in the regulation of transcription (Overvoorde et al., 2005). IAA33 was strongly expressed in the “B2” line and contained nucleotides change in the first exon (Figures 2, 3). This suggests that IAA33 plays a role in observing the differences of plant growth between the two parental lines. AIR encodes a protein related to a large family of proteins consisting of proline-rich or glycine-rich N-terminus and a hydrophobic region (Neuteboom et al., 1999); they are involved in responses to auxin, root development, and root hair cell differentiation. The expression of RAIR1 was slightly higher in “835” than in “B2,” suggesting RAIR1 may be involved in the root development of “835.” Regarding ARF19, auxin response factors are reported to bind auxin response promoter elements and mediate transcriptional responses to auxin, this gene functions in root development and primary root developmental processes (Wilmoth et al., 2005). Root growth and development are important for radish plant to acquire underground water and nutrients. Root development is determined by a combination of three major cell biological processes: the rate of cell division, the rate of cell differentiation, and the extent of expansion and elongation of cells (Scheres et al., 2002).

*The GA20OX3* gene is a member of the gibberellins gene family; these plant hormones are involved in controlling diverse aspects of growth and development (Hay et al., 2002; Yamaguchi, 2008). SPL9 encodes a putative transcriptional regulator involved in the vegetative to reproductive phase transition, and the regulation of leaf formation. Recently, BrpSPL9 (*B. rapa* SPL9) was reported to control the earliness of leaf heading time in Chinese cabbage (Aukerman and Sakai, 2003; Wang et al., 2014). As their expression levels were low in “835” and “B2” (Figure 3), these genes might not contribute to the differences observed during the plant growth. Furthermore, in *Cardamine hirsuta*, flowering locus C (ChFLC) alleles show both early flowering and affects leaf form by influencing the rate of age-dependent progression of development, including faster leaflet production (Cartolano et al., 2015). So the mechanisms of leaf or root development with reproduction are complex not limited to growth hormone and regulated factors, suggesting the need for further verification in radish study.

### QTL blocks are evolutionarily conserved between the *brassica* species

**Figure 4:**
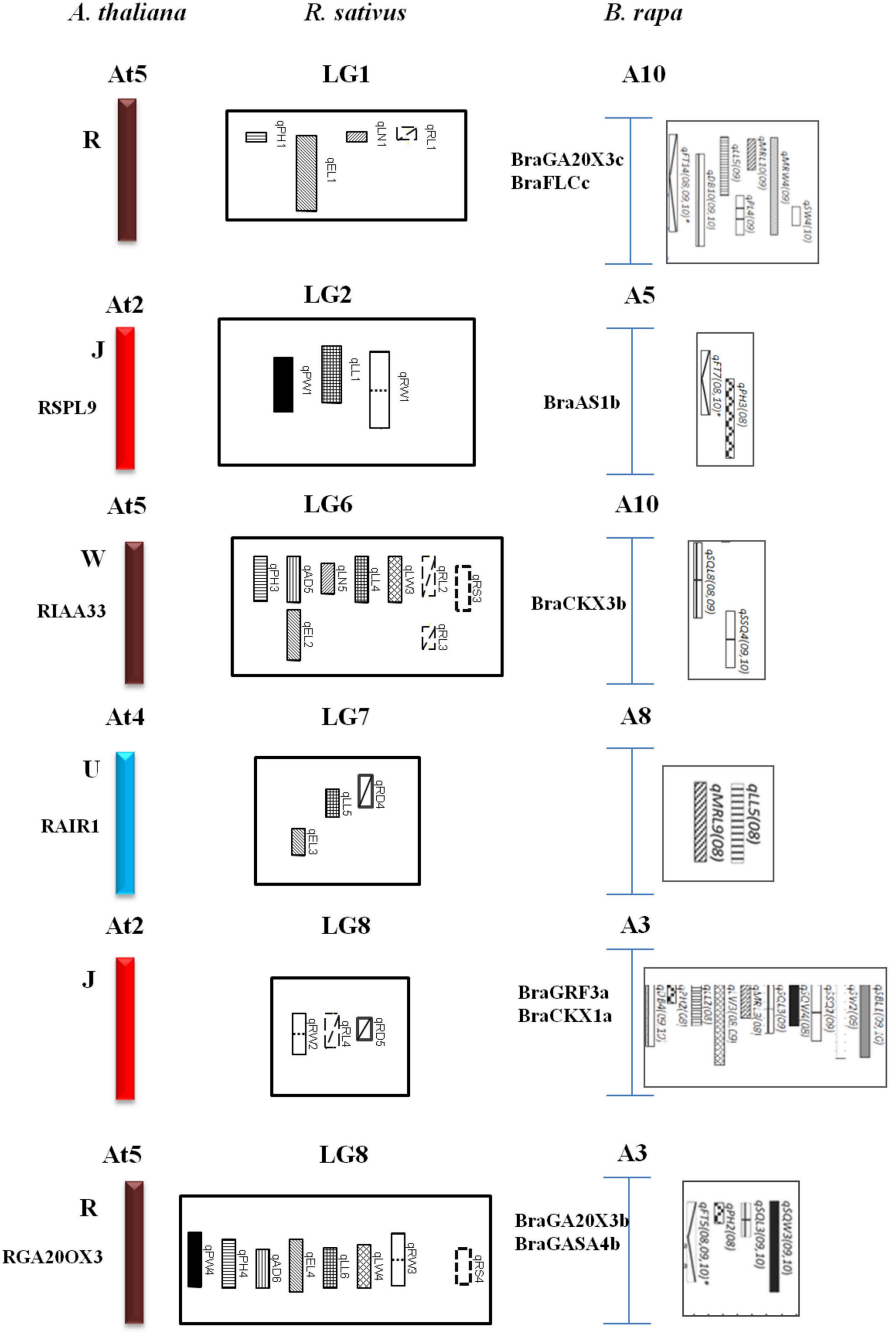
**Comparative QTL mapping between radish, *A. thaliana* and *B. rapa* revealed conservation of crucifer building blocks governing morphological traits**.

The comparative QTL map alignment between *A. thaliana* and *B. rapa* revealed conservation of QTL blocks containing several genetic loci influencing plant height, leaf length and leaf width between the *B. rapa* and radish (Figure 4). Although numerous studies have reported the conservation of chromosomal blocks in many crops (Parkin et al., 2005; Schranz et al., 2006; Panjabi et al., 2008), a comparison of QTL location have not been performed for morphological traits in radish genomes. In previous studies, Li et al. (2013) identified conserved QTL blocks in four of the 10 homologous groups of A, B, and C subgenomes. The R block, W block, and J and F blocks were major QTL blocks harboring common QTLs between A, B, and C subgenomes of these species (Li et al., 2013). In this study, conservation of these traits was investigated in the four homologous group QTL blocks. The R blocks, J block, W block, and U block were major QTL blocks harboring common QTL between the A genomes (A3, A5, A8, A10) and *A. thaliana* (At2, At4, At5). The first homologous group consisted common markers (cnu_BRPGM0092 and At5g7) in QTL area of radish LG1 and *B. rapa* A10, many major QTLs for different traits were detected in *B. rapa*, such as leaf traits for midrib length and midrib width, these are related to angular divergence and leaf growth traits in radish. Similarly, in third homologous group, two linkage markers (At4g45 and cnu_m090a) were both loci in this QTL region of radish and *B. rapa*. The second homologous group contained different functional genes: RSPL9 in radish LG2 and BraAS1b in *B. rapa* A5. Furthermore, the functional gene of GA20OX3 (R block) was detected at the same QTL position region of *B. rapa* and radish. This suggested gene governing these traits were structurally and functionally conserved, not only between the A, B, and C subgenomes of *Brassica* species but also between radish genomes, even though they diverged from their common ancestor a long time ago.

In conclusion, 55 QTLs for morphological traits are detected in radish genetic map and identified the candidate genes co-localization with important QTLs, but association analysis using large segregating population and functional validation of candidate genes by transformation should be done in further functional research. Whole-genome sequencing of the parental lines “835” and “B2” allowed the development of polymorphism-based molecular markers to generate a high-density map in different radish populations would give more information about the conservation and diversification of genetic loci governing all kinds of traits. However, we believe that the identified candidate genes for morphological traits can provide useful markers for breeding schemes in the future.

## Author contributions

*XY* and *SC* carried out the marker development, genetic map and QTL; data analyzed and wrote the manuscript. VD participated in sequencing analysis and sequencing assembles. JR carried out morphological traits survey. *JL, XL, and WP* modified the manuscript. *YL* conceived the study, participated in its coordination, and helped to draft the manuscript.

### Conflict of interest statement

The authors declare that the research was conducted in the absence of any commercial or financial relationships that could be construed as a potential conflict of interest.

## Supplementary material

Supplementary Table 1 **Marker information and chromosome number in *B. rapa* and homologuous loci in *A. thaliana* number**.

Supplementary Table 2 **Phenotypic correlations among the 11 traits in 2012, 2013, and 2014**.

Supplementary Table 3 **Gene-based markers sequence information and their respective homologs gene information in *Arabidopsis***.

Supplementary Figure 1 **Frequency distributions of leaf and root associated traits in the F_2:3_ population grown in 3-year field trials**. Solid arrows indicate “835” and diamond arrows indicate “B2.”
