# Supplementary material for: Quantitative Trait Loci for Morphological Traits and their Association with Functional Genes in Raphanus sativus
Source: Front Plant Sci. 2016 Mar 4;7:255. doi: 10.3389/fpls.2016.00255 (PMC4777717; doi:10.3389/fpls.2016.00255)
Supplement: Supplementary Table 2 — Phenotypic correlations among the 11 traits in 2012, 2013, and 2014. [file Table2.docx]

Supplementary Table 2. Phenotypic correlations among the 11 traits in 2012, 2013 and 2014.

|  | PW2012 | PH2012 | AD2012 | EL2012 | LN2012 | LL2012 | LW2012 | RW2012 | RL2012 | RD2012 | RS2012 |
| --- | --- | --- | --- | --- | --- | --- | --- | --- | --- | --- | --- |
| PW2012 | 1 |  |  |  |  |  |  |  |  |  |  |
| PH2012 | 0.731** | 1 |  |  |  |  |  |  |  |  |  |
| AD2012 | 0.721** | 0.887** | 1 |  |  |  |  |  |  |  |  |
| EL2012 | 0.705** | 0.712** | 0.663** | 1 |  |  |  |  |  |  |  |
| LN2012 | 0.573** | 0.637** | 0.655** | 0.468** | 1 |  |  |  |  |  |  |
| LL2012 | 0.702** | 0.906** | 0.919** | 0.641** | 0.575** | 1 |  |  |  |  |  |
| LW2012 | 0.722** | 0.861** | 0.910** | 0.650** | 0.607** | 0.911** | 1 |  |  |  |  |
| RW2012 | 0.933** | 0.701** | 0.693** | 0.748** | 0.503** | 0.669** | 0.676** | 1 |  |  |  |
| RL2012 | 0.753** | 0.855** | 0.774** | 0.844** | 0.531** | 0.779** | 0.753** | 0.770** | 1 |  |  |
| RD2012 | 0.640** | 0.651** | 0.738** | 0.515** | 0.582** | 0.720** | 0.765** | 0.642** | 0.602** | 1 |  |
| RS2012 | 0.501** | 0.601** | 0.599** | 0.665** | 0.412** | 0.588** | 0.558** | 0.547** | 0.729** | 0.456* | 1 |

|  | PW2013 | PH2013 | AD2013 | EL2013 | LN2013 | LL2013 | LW2013 | RW2013 | RL2013 | RD2013 | RS2013 |
| --- | --- | --- | --- | --- | --- | --- | --- | --- | --- | --- | --- |
| PW2013 | 1 |  |  |  |  |  |  |  |  |  |  |
| PH2013 | 0.805** | 1 |  |  |  |  |  |  |  |  |  |
| AD2013 | 0.765** | 0.902** | 1 |  |  |  |  |  |  |  |  |
| EL2013 | 0.672** | 0.821** | 0.746** | 1 |  |  |  |  |  |  |  |
| LN2013 | 0.720** | 0.807** | 0.788** | 0.664** | 1 |  |  |  |  |  |  |
| LL2013 | 0.758** | 0.912** | 0.946** | 0.752** | 0.755** | 1 |  |  |  |  |  |
| LW2013 | 0.732** | 0.876** | 0.908** | 0.723** | 0.726** | 0.956** | 1 |  |  |  |  |
| RW2013 | 0.934** | 0.744** | 0.711** | 0.606** | 0.619** | 0.716** | 0.675** | 1 |  |  |  |
| RL2013 | 0.758** | 0.835** | 0.743** | 0.606** | 0.675** | 0.761** | 0.749** | 0.754** | 1 |  |  |
| RD2013 | 0.782** | 0.796** | 0.833** | 0.660** | 0.750** | 0.860** | 0.841** | 0.757** | 0.671** | 1 |  |
| RS2013 | 0.407* | 0.658** | 0.596** | 0.461* | 0.547** | 0.601** | 0.603** | 0.391* | 0.763** | 0.455* | 1 |

|  | PW2014 | PH2014 | AD2014 | EL2014 | LN2014 | LL2014 | LW2014 | RW2014 | RL2014 | RD2014 | RS2014 |
| --- | --- | --- | --- | --- | --- | --- | --- | --- | --- | --- | --- |
| PW2014 | 1 |  |  |  |  |  |  |  |  |  |  |
| PH2014 | 0.518** | 1 |  |  |  |  |  |  |  |  |  |
| AD2014 | 0.541** | 0.524** | 1 |  |  |  |  |  |  |  |  |
| EL2014 | 0.372** | 0.378** | 0.387** | 1 |  |  |  |  |  |  |  |
| LN2014 | 0.477** | 0.252** | 0.416** | 0.300** | 1 |  |  |  |  |  |  |
| LL2014 | 0.518** | 10.000** | 0.524** | 0.378** | 0.252** | 1 |  |  |  |  |  |
| LW2014 | 0.550** | 0.623** | 0.540** | 0.362** | 0.382** | 0.623** | 1 |  |  |  |  |
| RW2014 | 0.894** | 0.419** | 0.411** | 0.268** | 0.325** | 0.419** | 0.359** | 1 |  |  |  |
| RL2014 | 0.594** | 0.328** | 0.304** | 0.696** | 0.305** | 0.328** | 0.267** | 0.553** | 1 |  |  |
| RD2014 | 0.838** | 0.443** | 0.447** | 0.221** | 0.309** | 0.443** | 0.481** | 0.816** | 0.325** | 1 |  |
| RS2014 | 0.679** | 0.060** | 0.207* | 0.691* | 0.959** | 0.050* | 0.270* | 0.515** | 0.679** | 0.300* | 1 |

| ** Correlation is significant at the 0.01 level (2-tailed) |
| --- |
| * Correlation is significant at the 0.05 level (2-tailed) |
