# Supplementary figures and images for: Quantitative Trait Loci for Morphological Traits and their Association with Functional Genes in Raphanus sativus
Source: Front Plant Sci. 2016 Mar 4;7:255. doi: 10.3389/fpls.2016.00255 (PMC4777717; doi:10.3389/fpls.2016.00255)

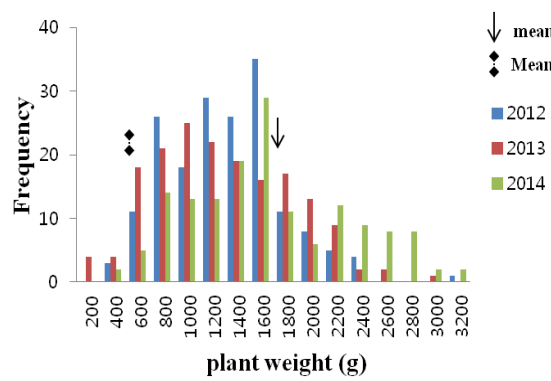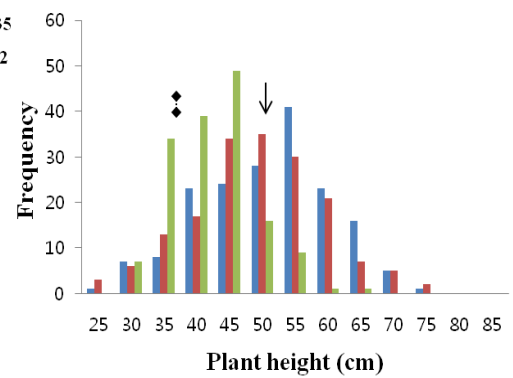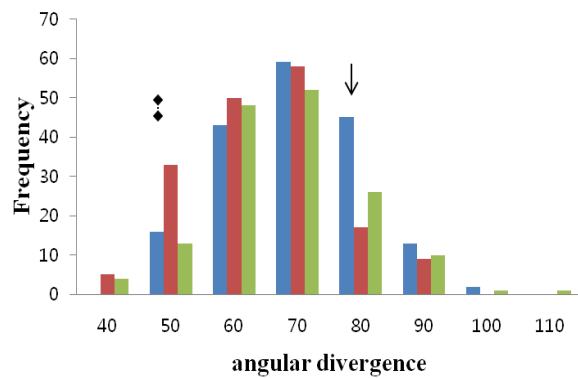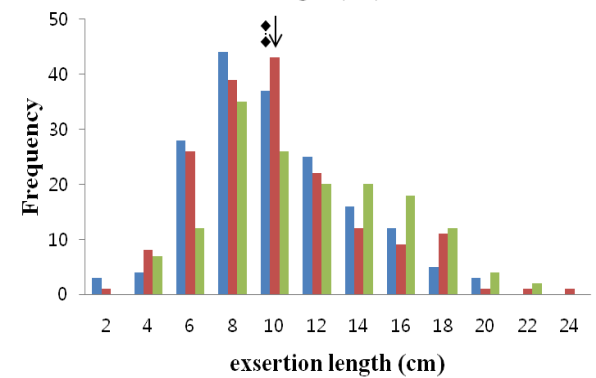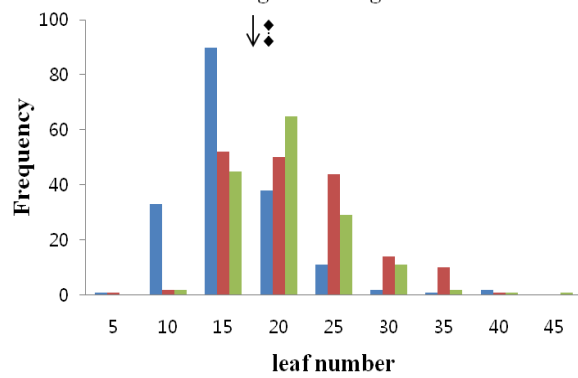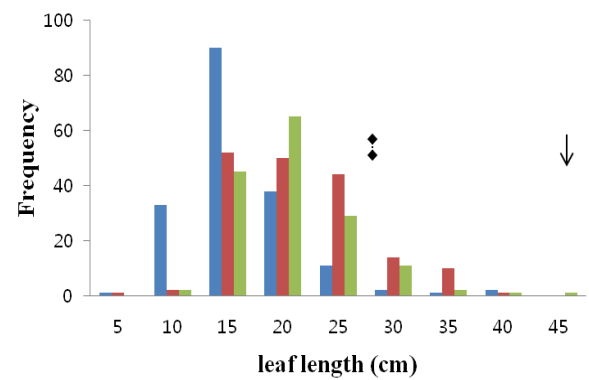

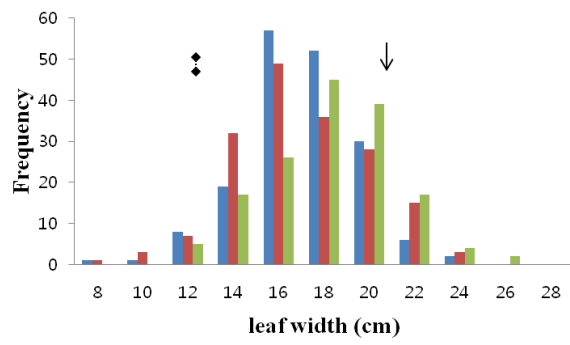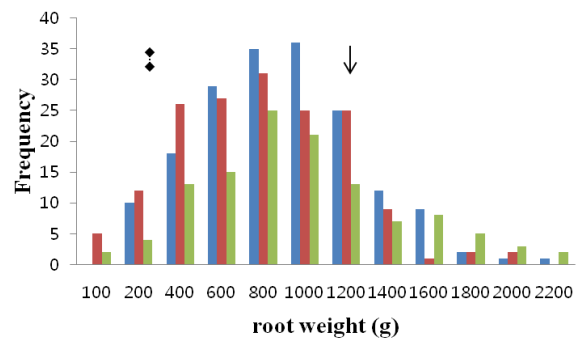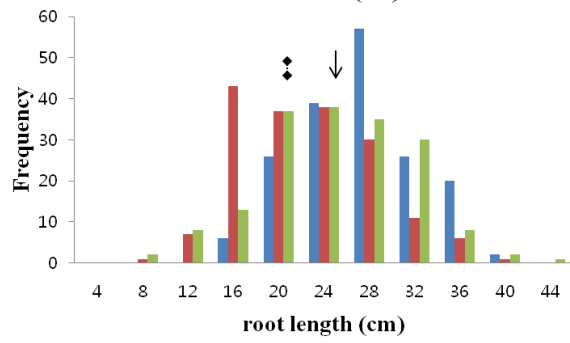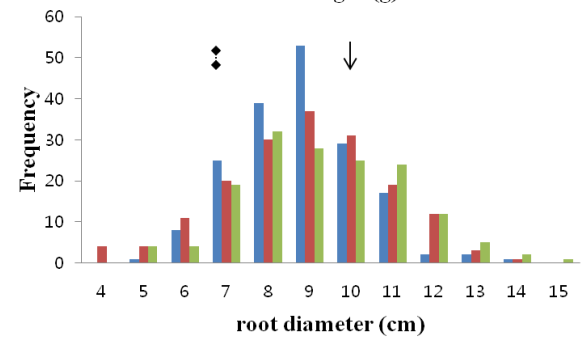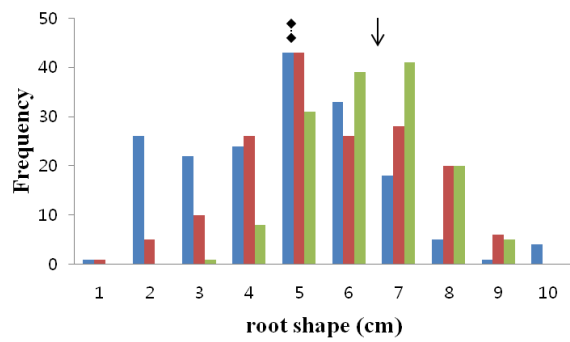

Supplement: Supplementary Figure 1 — Frequency distributions of leaf and root associated traits in the F2:3 population grown in 3-year field trials. Solid arrows indicate “835” and diamond arrows indicate “B2.” [file Image1.pdf]
